# Bioavailable Selenium Concentration and Bioavailability in Tissues of Beef Cattle

**DOI:** 10.3390/ani14223210

**Published:** 2024-11-08

**Authors:** Marta Juszczak-Czasnojć, Małgorzata Bąkowska, Dariusz Gączarzewicz, Bogumiła Pilarczyk, Agnieszka Tomza-Marciniak

**Affiliations:** Department of Animal Reproduction Biotechnology and Environmental Hygiene, West Pomeranian University of Technology, Janickiego 29, 71-270 Szczecin, Poland; malgorzata.bakowska@zut.edu.pl (M.B.); dariusz.gaczarzewicz@zut.edu.pl (D.G.); bogumila.pilarczyk@zut.edu.pl (B.P.); agnieszka.tomza-marciniak@zut.edu.pl (A.T.-M.)

**Keywords:** bioavailable selenium, tissues, beef cattle, intensive farming, mineral deficiencies

## Abstract

The aim of the study was to compare the levels of bioavailable Se between different organs in beef cattle with regard to selenium status. The animals were divided into two groups based on serum selenium concentration, viz. normal and deficient Se status. Bioavailable selenium was extracted from the organs by simulated in vitro digestion, and its concentration was determined by spectrofluorimetry. The percentage of bioavailable Se was calculated in the liver, kidney, lung, spleen, heart, and longissimus dorsi muscle, based on the total Se content. In all animals tested, the highest bioavailable selenium concentrations were found in the kidney (0.175–0.408 µg/g) and the lowest in the longissimus dorsi muscle (0.04–0.042 µg/g), regardless of Se status. The highest bioavailability of Se was recorded in bulls, reaching 56.6% in the longissimus dorsi muscle, and the lowest in the kidney (21.4%). The study showed that cattle with Se deficiency had a higher percentage of bioavailable Se in their tissues than those with normal Se levels. This is most likely related to the fact that the body seeks to ensure optimal levels of the element in the organs during insufficient supply. Despite balanced feeding, there was a deficiency of Se. This is most likely related to the fact that, in plants, it occurs mainly in its inorganic form, which is less well-utilised by the animals and does not cover their needs for this element.

## Simple Summary

Selenium is an essential element in the diet of both humans and animals. By obtaining adequate levels of Se in the animal body, animal products can be obtained that will provide a valuable source of Se in the human diet. The present study investigated the bioavailable selenium content in organs and tissues from beef cattle. Our results show that selenium-deficient animals had a higher percentage of bioavailable selenium in tissues than animals with normal selenium levels. In addition, the highest concentration of bioavailable selenium was found in the kidneys and the highest percentage of bioavailable selenium was found in the muscles. The results of the study indicate that more attention should be paid to selenium supplementation in animals to ensure normal selenium levels during insufficient supply.

## 1. Introduction

The selenium (Se) content of the soil varies considerably between regions due to the type of bedrock in the area and the way the soils are used. In Europe, soils are generally poor in Se [1]. In Poland, more than half of the country is affected by Se deficiency [2], with the Se concentration ranging from 0.040 to 0.640 µg/g d.m. [3]. The soil–plant/feed–animal transfer ratio is estimated to be 1:0.36:0.14 for organic farms and 1:0.26:0.24 for conventional farms [4].

The final amount of Se in plants that can be utilised by animals results not only from its total content in the plant, but also from inter alia the chemical form of the element and the presence in the diet of compounds that may increase or decrease the bioavailability of selenium. In particular, selenium absorption from the gastrointestinal tract of animals is limited by dietary sulphur or cyanogenic glycosides; the latter are commonly found in legumes, which are a component of animal mixtures [5]. Selenium is readily absorbed from a protein-rich diet and in the presence of vitamins A and E [6,7,8,9]. Vitamin E and Se act as antioxidants in the ruminant body; they also positively affect overall homeostasis and, importantly, improve animal health and performance by reducing heat stress [10].

The selenium present in food occurs mainly in its organic forms, as selenocysteine (SeCys) and selenomethionine (SeMet). The inorganic forms of Se, such as sodium selenite and sodium selenate, are present only in small amounts. The main form of selenium that occurs in plants, and thus in ruminant feed, is selenomethionine (SeMet). As the animal metabolism is unable to distinguish between SeMet and methionine, SeMet is incorporated into animal proteins in place of methionine; similarly, selenocysteine is incorporated instead of cysteine [6,11].

Selenium absorption mainly takes place in the small intestine. This absorption and its metabolism are primarily influenced by the concentration and chemical form of Se in the diet, but they are also affected by the levels of other dietary components [12,13]. The bioavailability of Se varies considerably depending on its chemical form. For example, organic Se is absorbed more effectively than inorganic Se by both monogastric animals and ruminants. Unfortunately, most plant-based livestock feed is low in Se and is supplemented with inorganic Se. While 80% of organic Se is absorbed in monogastric animals and poultry, only 29% is taken up by cattle [5,14]; in contrast, up to 80% of organic forms are absorbed, mainly SeMet and SeCys [13]. In addition, organic Se is retained for longer in the body than inorganic Se (SeO_4_^2−^ or SeO_3_^2−^), resulting in better utilisation [15].

In ruminants, a key role in Se bioavailability is played by the microorganisms of the rumen; these reduce inorganic sodium selenite to unavailable elemental Se or selenides, thus lowering its bioavailability and increasing its excretion in faeces [16]. Rumen bacteria also incorporate organic SeMet about four times more readily than inorganic Se, and organic Se undergoes fewer changes in the rumen than inorganic Se, facilitating better absorbance. Organic selenium stimulates the microorganisms of the digestive tract in a dose-dependent manner, and influences rumen fermentation and feed digestion, which is reflected inter alia in milk yield. Although the mechanism of Se absorption in the rumen remains unclear, it is known that it begins with rumen microorganisms in the digestive tract, and that its breakdown is mediated by lysozyme. The protein produced by microorganisms has a high biological value, allowing Se to be easily absorbed in the small intestine [16,17]. The mechanism of absorption in the small intestine is similar in ruminants and monogastric animals [5]. In ruminants, Se is first transformed in the rumen, from which it is either absorbed directly or passes along to the small intestine, the main site of digestion and absorption; it is estimated that about 60% is absorbed in this location [18].

SeMet and SeCys are absorbed in the duodenum, and inorganic Se in the ileum. 40% of SeMet not absorbed in the rumen is absorbed in the small intestine at a rate of 80% [13,19].

The Se content of individual bovine tissues varies considerably and depends mainly on the form of Se supplied by the diet. During supplementation, part of the absorbed organic Se is incorporated directly into muscle proteins; in contrast, inorganic Se is mainly used for selenoprotein synthesis and does not influence muscle Se concentration [20]. The Se content of meat also varies between species due to differences in the structure and function of the digestive organs and, consequently, their selenium metabolism [5,16]. In addition, Se supply also influences its content in animal tissues and organs: if the supply is too low, the Se level in food products, i.e., eggs, milk and meat, will also be low. Liver Se level is considered an indicator of overall Se status for the animal. Zhang et al. [21] report that supplementation with both SeNa_2_ and SeMet increases Se levels in tissues and organs, but to different degrees. Animals receiving SeMet demonstrated significantly higher Se concentrations in the serum, semitendinosus muscle, heart, liver, and kidney.

By obtaining adequate levels of Se in the animal body, animal products can be obtained that will provide a valuable source of Se in the human diet. The concentration of bioavailable Se in tissues from fish is already well understood, whereas in the case of livestock and, in particular, tissues used for consumption from cattle, the determination of Se concentrations requires further research [22]. Therefore, the aim of the present study was to determine the bioavailable Se (Se_B_) content in tissues and organs of beef cattle used for consumption.

## 2. Materials and Methods

The research material was collected at a slaughterhouse in the north of Poland with the permission of the District Veterinary Officer. The collection of tissues took place during two visits to the slaughterhouse. As the animals were not slaughtered for research purposes, the approval of the Bioethics committee was not needed. These principles are in line with the Local Ethics Committee [23].

A veterinarian examined the animals before slaughter and assessed them as healthy. Immediately after slaughter, organ samples were taken; all had been previously assessed by a veterinarian and declared fit for consumption. The study material consisted of blood, liver, kidney, lung, spleen, heart, and longissimus dorsi muscle. The blood sample collection was taken during exsanguination at the slaughterhouse (shortly after killing). The Se status of each animal was determined based on serum Se concentration, and the animals were divided into two groups, optimal (0.08–0.3 µg/mL) and below-optimal Se levels (below 0.08 µg/mL), as in accordance with Puls [24]. For a better visualisation of the results the minimum optimal level of Se was determined according to the criteria described by Puls [24].

Due to differences in diet, the technology groups were not compared with each other. The technology groups were divided by sex and females were further divided by age.

### 2.1. Animals

All cattle were kept in a production herd on a conventional semi-intensive farm. Material for the study was collected during winter feeding. On the farm, animals undergo ongoing body condition assessments according to the BCS 5-point assessment. All animals used in the study were characterised by optimal body condition (BCS 3). Each technology group of cows (*n* = 24, mean age 83 months, range 50–146 months), heifers (*n* = 16, mean age 22 months, range 19–24 months), and bulls (*n* = 23, mean age 23 months, range 21–25 months) was fed according to the standards adopted by the farm. The total amount of Se consumed by the animals consisted of Se contained in the plant feed and that contained in the mineral feed. The chemical profile of the feed is shown in Table 1.

### 2.2. Chemical Analyses

#### 2.2.1. Se Determination

Bioavailable selenium was extracted from the tissues and organs by simulated in vitro digestion. The procedure used a mixture of hydrochloric acid, pepsin (6% *m*/*v* pepsin in 0.15 mol/L HCl), and a pancreatin-bile-alpha amylase mix (1.5% *m*/*v* pancreatin, 0.15% *m*/*v* bile, and 0.5 alpha amylase in 0.15 mol/L NaCl) as described by Cabañero et al. [22]. The extract was then subjected to wet digestion with concentrated nitric acid V and perchloric acid VII according to Pilarczyk et al. [25], and the level of Se was determined by spectrofluorimetry using 2,3-diaminonaphthalene. All chemicals were of analytical reagent grade.

The percentage of bioavailable Se was given according to the total Se content of each organ [22].

The uptake of Se from the tissues/organs of beef cattle, i.e., its bioavailability, was determined according to the following formula:

(1) $$Bioavailability=\frac{Se\;concentration\;in\;digested\;extract\;-\;Seconcentration\;in\;digestion\;reagents}{Total\;Se\;concentration\;in\;tissue\;or\;body}\times 100\%$$

#### 2.2.2. Validation of Results

The present work used two certified reference materials to validate its methodologies: Seronorm (Trace Elements Serum L-1) (Sero AS, Billingstad, Norway) and BCR 185R (bovine liver). Seronorm achieved 95.2% of mean Se reference values and BCR 185R (bovine liver) 94.2%.

#### 2.2.3. Statistical Analysis

Statistical analysis was performed using Statistica software (StatSoft Inc., ver. 13.3 StatSoft, Tulsa, OK, USA). The Shapiro–Wilk test was used to confirm the normality of the distribution. Variables with non-normal distribution were adjusted to normal distribution using logarithmic transformation (log10) and subjected to one-factor analysis of variance (ANOVA). Significant differences were determined using Duncan’s test. Differences with a *p*-value < 0.05 were considered statistically significant. The minimum sample size was calculated according to the Cochran formula. The data were stored in the University’s repository.

## 3. Results

### 3.1. Se_B_ Concentration

#### 3.1.1. Se_B_ Concentration in Cows

**Figure 1:**
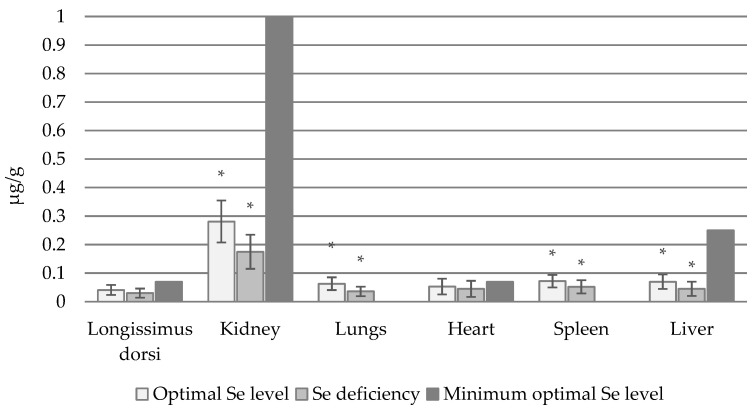
Se_B_ concentration in individual organs (µg/g) of cows with normal and deficient Se status (*—statistically significant differences (*p* < 0.05) in Se_B_ concentration in a given organ between animals with normal and Se-deficient status).

In cows, the lowest Se_B_ concentration was recorded in the longissimus dorsi muscle: 0.041 µg/g in animals with optimal Se concentrations and 0.03 µg/g b.w. in those with Se deficiency (Figure 1). In contrast, the highest Se_B_ concentrations were recorded in the kidneys of both normal and Se-deficient animals.

#### 3.1.2. Se_B_ Concentration in Bulls

**Figure 2:**
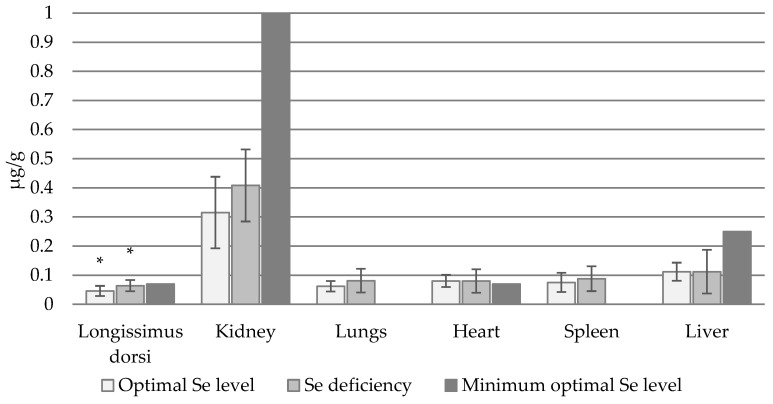
Se_B_ concentration in individual organs (µg/g) of bulls with normal and deficient Se status (*—statistically significant differences (*p* < 0.05) in Se_B_ concentration in a given organ between animals with normal and Se-deficient status).

The highest Se_B_ concentrations were recorded in the kidney: 0.315 µg/g in bulls with normal Se status and 0.408 µg/g in those with Se deficiency. In contrast, the lowest Se_B_ concentrations were found in the latissimus dorsi muscle (Figure 2). The two groups generally demonstrated similar Se_B_ concentrations in individual organs (*p* > 0.05), with the exception of the longissimus dorsi muscle, where significantly higher levels were found in Se-deficient animals.

#### 3.1.3. Se_B_ Concentration in Heifers

**Figure 3:**
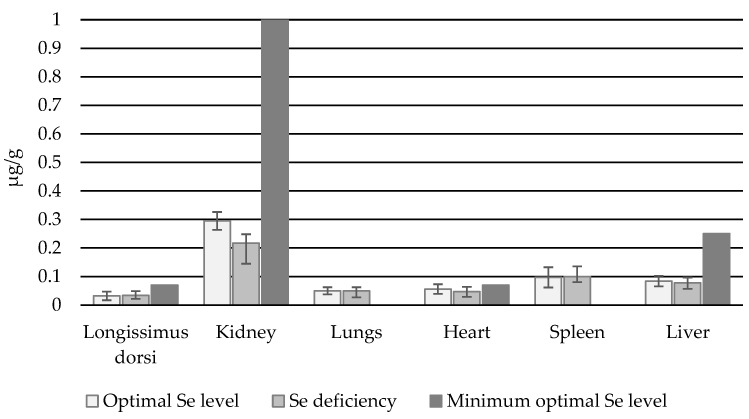
Se_B_ concentration in individual organs (µg/g i.m.) of heifers with normal and deficient Se status.

In heifers, the highest Se_B_ concentrations were recorded in the kidneys, this being several times higher than in other organs. The lowest Se_B_ concentration was found in the latissimus dorsi muscle: 0.032 µg/g in the normal group and 0.034 µg/g in Se-deficient animals (Figure 3). In both the normal and deficient groups, the muscle demonstrated three times lower Se_B_ content than the spleen, and approximately 2.5 times lower content than the liver (*p* < 0.05).

### 3.2. Bioavailability of Se in Tissues of Beef Cattle

#### 3.2.1. Cows

**Figure 4:**
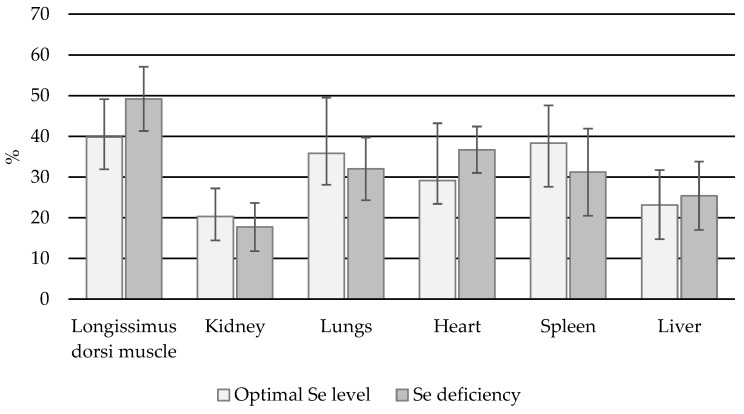
Percentage share of Se_B_ with regard to total Se content in individual organs in cows.

In the cows, the % bioavailable Se in muscle, heart, and liver tissue increased as selenium status decreased (Figure 4). No significant differences in % bioavailable Se were found between the normal and Se-deficient groups (*p* > 0.05).

#### 3.2.2. Bulls

**Figure 5:**
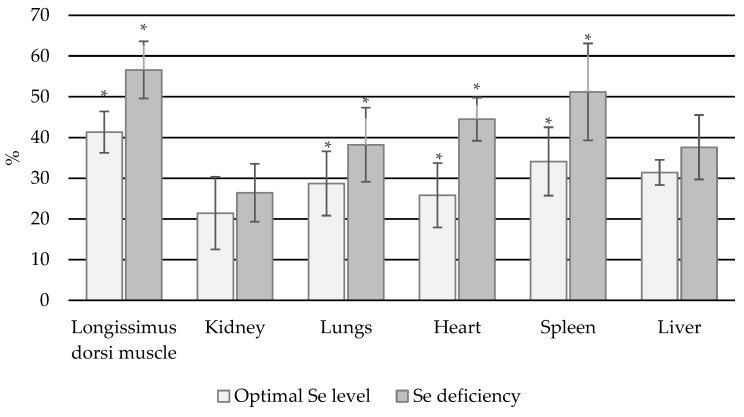
Percentage of Se_B_ in total Se content in individual organs in bulls (*—statistically significant differences (*p* < 0.05) in a given organ between animals with normal and Se-deficient status).

In all bull organs, higher % Se bioavailability was associated with lower Se status. Significant differences in % bioavailable Se content were found between the normal and deficient groups with regard to the longissimus dorsi muscle, lungs, heart, and spleen (*p* < 0.05) (Figure 5).

#### 3.2.3. Heifers

**Figure 6:**
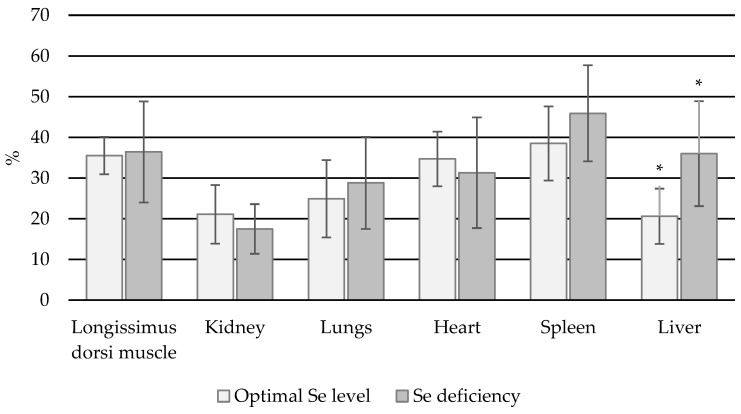
Percentage of Se_B_ in total Se content in individual organs in heifers (*—statistically significant differences (*p* < 0.05) in a given organ between animals with normal and Se-deficient status).

In the muscle, lung, spleen, and liver, higher % bioavailable Se was associated with lower Se status (Figure 6). When comparing the % Se bioavailable in animals with normal and deficient levels of total Se, statistically significant differences were observed only for the liver (*p* < 0.05).

## 4. Discussion

The Se content of animal products varies depending on the type of tissue tested, the species and breed of animal, how they are fed and how the food products are processed. The maturation period of meat can also play a role, with Se bioavailability falling over time [26,27].

Both humans and animals have access to only a portion of the total Se content of the diet, as only a certain pool of this element exists in a bioavailable form [27,28]. The bioavailability of Se, i.e., the true amount that can be absorbed and used by the body, is mainly determined by its chemical form; in animal tissues, most Se exists as organic compounds [29,30,31]. These include the selenoamino acids SeMet and SeCys, which are derived from the digestion of Se-containing proteins, selenosaccharides, and the methyl metabolites of Se metabolism: TMSe+. These are accompanied by selenides, which are inorganic products of selenoamino acid catabolism [32].

Our findings indicate that most organs in Se-deficient animals had a higher % Se_B_ compared to those with normal Se levels. This may indicate that their pool of poorly bioavailable Se compounds, e.g., selenides and SeCys, is reduced during Se deficiency. This decrease in may be due to reduced catabolism of selenoamino acids, their utilisation for the synthesis of priority selenoproteins [32], or the redistribution of metal selenides to organs playing a role in their metabolism [22]. It is likely that the increase in the proportion of bioavailable compounds observed in the organs of Se-deficient animals, particularly those that bind to the organ tissue as structural proteins, was influenced by a decrease in the unavailable forms of Se.

The predominant organic Se compound in animal tissues is SeMet, which non-specifically substitutes methionine in muscle proteins [33,34]. SeMet is specifically incorporated into organs with a high rate of protein synthesis, such as skeletal muscle and the pancreas, liver and kidney [35]. This allows a stock of Se to accumulate in animal tissues, which can be utilised at times of increased selenoprotein demand [36].

The second most important selenoprotein present in animal tissues is SeCys, which, due to its lack of storage capacity and rapid excretion when unused, is a suitable marker for assessing the current Se intake from the diet [35,36,37]. A Se deficiency in the feed leads to a decrease in the SeCys content in the organs, which may also be reflected in a higher % of bioavailable Se forms. Although SeCys is an organic compound, it is less bioavailable than SeMet, and its Se utilisation efficiency is closer to the poorly bioavailable sodium selenate VI than SeMet [38].

In the present study, the mean bioavailability of Se in the studied organs ranged from around 10–20% to 56.6%, depending on the technology group. The highest bioavailability was found for the latissimus dorsi muscle and the lowest for the kidneys. These findings are lower than those reported by Ramos et al. [27] and Daun et al. [26], who found the bioavailability of Se in the latissimus dorsi to be 72–80%. Reeves et al. [39] note that increasing the dose of Se to animals results in an increase in Se concentrations in muscle and liver tissue, with the largest increase observed for SeMet.

In contrast, Martínez et al. [40] report the bioavailability of Se in poultry meat to be only 33.3%. However, the study was performed on Caco-2 cells, and the authors emphasise that data based on in vitro research does not reflect the complexity of the structure and function of the human digestive tract.

A study by Afonso et al. [41] showed Se to have high bioavailability in both raw (94%) and cooked fish meat (95%), with the least available form being grilled fish meat (82%). The high bioavailability of Se in fish meat has also been reported by other authors [22,42,43].

Interestingly, our data indicate that among the tested organs, the kidneys appeared to have the highest concentration of Se_B_ but also the lowest proportion of bioavailable forms of Se. This is probably explained by the accumulation of poorly absorbed metal selenides in the kidneys. A study on the effect of Se supplementation (sodium selenate IV) on Hg II bioaccumulation in chickens found the kidney to be the target organ for mercury selenide [44]; it is reasonable to assume that similar mechanisms would be used for accumulating other metals.

Se deficiency in cattle feed rations can cause a number of disease states with both clinical and subclinical courses. One of the most serious effects of Se deficiency in animals is reproductive problems. In cows, foetal necrosis and retention of the placenta after parturition can occur, and in males the quality of semen produced is reduced. Young animals show a weaker body condition and, as a result of the deficiency, there is an impairment in the formation of horn parts [45,46,47]. In older cows, Se deficiency may increase the risk of mastitis [48]. Se deficiency also causes muscular dystrophy. Due to changes in the cardiac muscle, sudden death of the animal can occur [49]. Farmers should monitor the Se intake of their animals and, if a deficiency of this element is found in cattle, increase the proportion of organic forms of Se in the ration. The most common method of supplementing the diet with essential micronutrients, including Se, is the use of salt licks [50]. However, the administration of Se via salt licks is not entirely accurate, due to the fact that it is not known how much actual Se the animal has taken in. Another form of Se enrichment in the diet is to add it to energy and protein feeds. Selenium in mineral feed is present in both organic and inorganic forms [19]. Selenium can also be added to feed in the form of selenium yeast, in which it occurs in organic form [51]. A direct method of Se administration is the use of sodium selenite boluses. These are characterised by a prolonged effect, lasting up to 6 months. Using a special syringe, the bolus is placed in the rumen, ensuring that the entire dose is used [50].

## 5. Conclusions

In general, all animals showed low Se concentrations in the longest dorsal muscle. This may be due to the fact that, in animals, the highest Se retention rate is found in the reproductive organs, which have a priority in the uptake of this element. Cattle with Se deficiency demonstrated a higher percentage of bioavailable Se in their total tissue Se content than those with normal Se levels. The highest bioavailability was recorded in the longissimus dorsi muscle, which was 56.6% in bulls and 49.2% in cows. In heifers it was 45.9%. This is most likely due to the need to ensure optimal levels of Se in the organs during insufficient supply. Farmers should monitor the intake of Se by their animals and increase the proportion of organic Se in the feed in the event of deficiency. This will produce a feed rich in Se with high bioavailability.

## Figures and Tables

**Table 1:** The total chemical composition of the feed (mean ± standard deviation).

Animal	Dry Matter (g)	Crude Protein (g)	Raw Ash (%)	Raw Fibre (g)	Metabolic Energy (MJ)	Selenium (mg)
Cows	10,200 ± 158.1	802.1 ± 68.7	6.34 ± 0.35	1628.2 ± 111.2	93.28 ± 14.6	3.06 ± 0.02
Heifers	8370 ± 103.4	761.3 ± 54.5	5.95 ± 0.51	1524.7 ± 95.8	79.24 ± 7.4	3.08 ± 0.05
Bulls	11,900 ± 115.8	1331.2 ± 98.3	8.29 ±0.07	2387.2 ± 167.7	115.74 ± 19.2	2.52 ± 0.1

## Data Availability

All data generated and analysed during this study are included in this published article. Raw data supporting the findings of this study are available from the corresponding author on request.
